# Vitamin D and Allergic Disease: Sunlight at the End of the Tunnel?

**DOI:** 10.3390/nu4010013

**Published:** 2011-12-28

**Authors:** Anderson P. Jones, Meri K. Tulic, Kristina Rueter, Susan L. Prescott

**Affiliations:** School of Paediatrics and Child health, University of Western Australia, Perth, Western Australia 6008, Australia; Email: apjones@meddent.uwa.edu.au (A.P.J.); mtulic@meddent.uwa.edu.au (M.K.T.); kristina.rueter@health.wa.gov.au (K.R.)

**Keywords:** vitamin D, 25(OH)D, helper T cells, allergy, vitamin D receptor, immune system

## Abstract

A role for vitamin D in the regulation of immune function was first proposed after the identification of Vitamin D Receptors in lymphocytes. It has since been recognized that the active form of vitamin D, 1α,25(OH)2D3, has direct affects on naïve and activated helper T cells, regulatory T cells, activated B cells and dendritic cells. There is a growing body of literature linking vitamin D (serum 25(OH)D, oral intake and surrogate indicators such as latitude) to various immune-related conditions, including allergy, although the nature of this relationship is still unclear. This review explores the findings of epidemiological, clinical and laboratory research, and the potential role of vitamin D in promoting the inappropriate immune responses which underpin the rise in a broad range of immune diseases.

## 1. Introduction

Vitamin D is a secosteroid obtained either through endogenous production in the skin with exposure to UVB radiation, or from dietary sources. Within the body vitamin D acts as a hormone and is well recognized for its role in calcium and phosphorus homeostasis and skeletal health. We now know that all tissues in the body posses receptors for the active form of vitamin D, 1,25-dihydroxyvitamin D (1α,25(OH)2D3) and it has recently been discovered that there are extrarenal tissues capable of converting 25(OH)D to the active metabolite, including certain immune cells [1,2]. Aside from skeletal health, vitamin D has been associated with various cancers and autoimmune disorders [1] with evidence for a role in development and maintenance of lung structure and function [3,4,5]. It is this association with immune and airway function that provides the basis for the hypothesis that vitamin D may have direct links with asthma and allergic disease [6,7]. We aim to review the current evidence on vitamin D in relation to immune mechanisms and focus on the effect of pregnancy and early life exposure on allergy development.

## 2. Vitamin D in the Context of the Allergy Epidemic

The epidemic rise in allergic disease is now a major public health crisis, affecting more than 40% of the population in developed countries [8], with enormous impact on individuals, societies and health care systems [9]. The greatest burden of these diseases is during childhood, when the rapidly rising rates of disease are most evident [10]. As these younger generations reach maturity, it is possible that the impact of allergic disease will increase even further, especially as the same trends are emerging in developing countries undergoing lifestyle transition [10]. Furthermore, while the incidence of asthma appears to have reached a plateau in some developed nations, many of these regions are now facing a rise in food allergy in what appears to be a “second wave” of the allergy epidemic [11].

The rise in allergic disease is unequivocally linked to environmental and lifestyle factors associated with industrialization and progressive “Westernization”. A number of causative factors have been proposed for this including significant changes in lifestyle patterns, nutrient intake, exposure to microbial organisms and air pollution. However, most of these notions cannot individually explain the rise in disease. More recently variations in vitamin D status and intake has been implicated in allergy development and considered as one of a number of explanations for epidemiological and immunological associations. 

## 3. Is There Evidence of Vitamin D Deficiency with Lifestyle Change?

During the industrial revolution the general population experienced a decrease in exposure to solar radiation secondary to more urban living, indoor occupation and atmospheric pollution [12]. This resulted in a dramatic and overt rise in vitamin D deficiency as rickets became endemic during this period. While the incidence of rickets has been largely reduced in Western countries through the use of dietary supplementation and UV exposure [12], the same societies continue to experience a high prevalence of sub-clinical vitamin D deficiency or insufficiency [13] that is likely related to indoor lifestyle habits with limited sun exposure. Although often not significant in terms of rickets or osteomalacia, it remains unknown what, if any, are the clinical outcomes of vitamin D insufficiency. A large percentage of human vitamin D requirements are provided via sun exposure [14] (at latitudes with adequate UVB exposure) which acts through stimulating the conversion of 7-dehydrocholesterol into D3 in the skin [15]. Lifestyle patterns and environments that do not facilitate adequate sun exposure (indoor occupation, “screen time” and active sun avoidance) predispose to inadequate vitamin D synthesis and status. Such populations are therefore reliant on dietary or supplemental sources of vitamin D; a factor that may be playing a role in the current allergy epidemic [16,17]. 

The global rise in adult and childhood obesity [18] may also be linked to lower serum 25(OH)D. Serum 25(OH)D is inversely associated with body mass index (BMI) and adiposity in children [19,20] and adults [21]. This association persists after adjusting for physical activity and dietary intake suggesting obesity is an independent risk factor for reduced serum 25(OH)D and supports the notion that vitamin D deficiency may follow from modern lifestyle change. 

An area for concern is the recognition of deficiency and insufficiency in pregnant women globally [22]. The developing foetus depends entirely on its mother for supply of 25(OH)D and poor maternal vitamin D status is reflected in the infant at birth [23]. In non-supplemented infants serum 25(OH)D status tends to decrease over the first few months of life until sun exposure is increased and vitamin D-containing foods introduced into the diet. This raises the question of whether vitamin D status *in utero* and early life has effects on immune function and development of allergic disease.

## 4. Immunological Basis for the Vitamin D Hypothesis: Potential Mechanisms

### 4.1. Immune Cell Differentiation and Proliferation

The role of vitamin D in regulation of the immune system was first recognized through the identification of Vitamin D receptors on peripheral blood mononuclear cells (lymphocytes) [2]. It has since been discovered that 1α,25(OH)2D3 has direct affects on activated helper T cells [24] as well as regulatory T cells, activated B cells and dendritic cells (DC) [25]. Differentiation of naive helper T cells into major subgroups of effector T cells (namely Th1 or Th2 cells) or into regulatory T cells is dependent on the microenvironment to which they are exposed [2]. Some studies have shown that the presence of 1α,25(OH)2D3 inhibits macrophage production of IL-12 [26], decreases lymphoproliferation and production of IFN-γ and IL-2 from Th1 cells [2,27,28]. The resulting Th2 relative dominance has been used as an explanation for the earlier observation (1990’s) that the rise in allergic disease coincided with the introduction of routine vitamin D fortification in high latitude regions [16,17]. However, just as with the epidemiological associations, the effects of vitamin D on Th2 cells have also been contradictory, with reports of both enhancement and inhibition [29,30]. 

In animal models, an increased Th2 response to vitamin D has been accompanied by increase in anti-inflammatory IL-10 response [30]. In human studies there is some evidence of a non-linear response to vitamin D, in that both high and low levels have been associated with increased Th2 activity, but also evidence of an increased regulatory response. Accordingly, there is increasing evidence that the immunomodulatory effect of vitamin D may act not only through modulation of helper T cell function, but also through induction of CD4+CD25+ regulatory T cells (Tregs) [29]. This may be one possible explanation for the epidemiological links between both autoimmune disease and allergy with vitamin D deficiency [31]. 

Tregs include a diverse range of cells that appear to exert their effect variously through inhibiting transcription of the inflammatory cytokine IL-2, expressing IL-10, and potentially converting effector T cells to hyporesponsive or regulatory forms [30,32]. One of the factors shown to enhance Treg proliferation is the presence of the active form of vitamin D, 1α,25(OH)2D3, which binds to the retinoic acid receptor of naive T cells, inducing Treg differentiation [30]. To date, the majority of research has been conducted using cells sourced from peripheral blood; however some authors have proposed that gut-associated lymphoid tissue may be of relevance, particularly in regard to oral vitamin D intake and food allergen sensitization [33]. 

### 4.2. Gut-Associated Lymphoid Tissue

The intestine has evolved a highly specialized immune network to cope with vast exposure to an array of foreign antigens (from microbial flora and dietary products) at its luminal surface. This network (the GALT) is the largest immune “organ” of the body and comprises distinct tissues (such as the Peyer’s patches and mesenteric lymph nodes) as well as a vast array of migrating cells that passage through the local tissues (such as DC, B cells, effector T cells and a range of regulatory populations). These all act together to provide a finely balanced environment which affords immune defense against pathogens while at the same time suppressing unwanted inflammation against harmless commensals and allergens [34]. As oral tolerance is arguably one the most critical functions of the GALT, there is growing interest in the developmental events in the gut that could contribute to the inappropriate immune responses associated with rising rates of food allergy. Given that B and T cells are influenced by vitamin D (Section 4.1), there is speculation that orally ingested vitamin D could have the potential to directly modulate the function of lymphocytes in the local gut micro-environment. Weiss [33] proposes in his editorial on vitamin D and the gut microbiome that vitamin D could potentially interact directly with both the GALT and the microflora, to influence immune function and allergy outcomes, a theory also explored by Vassallo and Camargo [35]. In this publication the authors speculate that compromised antimicrobial production and modulation of host-microbe signaling pathways may alter the microbiome and predispose the host to infections. The “vitamin D/microbiome hypothesis” has proponents on both sides, with Vassallo and Camargo [35] arguing that deficiency is causative, while Wjst [17] suggests that the introduction of vitamin D supplementation coincides with the rise in allergic disease. This line of argument highlights that the potency and pharmacokinetics of endogenous and exogenous vitamin D may be different and that serum 25(OH)D, sun exposure and oral intake should be considered independently of one another.

### 4.3. Lung Structure and Function

In mice, Zosky *et al.* [4] found that vitamin D deficiency in mice is significantly associated with decreased lung volume, tissue volume and increased airway resistance. They proposed that, as reduced lung volume is a risk factor for lung function and respiratory illness in humans, vitamin D deficiency could increase the risk of these conditions through effects on lung tissue growth. There is some support for this in human studies linking vitamin D deficiency with reduced lung function [3,5]. Specifically, after adjusting for potential confounding factors (such as body size, gender, smoking history and physical activity), Black and Scragg [5] reported a significant association with serum 25(OH)D on forced expiratory volume (FEV) and forced vital capacity (FVC). This finding was recently confirmed in a Chinese population of newly diagnosed asthmatic adults [3]. The latter study found a high prevalence of vitamin D deficiency in patients, with 88.9% of having serum 25(OH)D <50 nmol/L. Forced expiratory volume, FEV as a percent of predicted, and FVC were all significantly positively correlated with serum 25(OH)D concentration (*p* < 0.05). 

The exact physiological mechanisms behind these observations remains unanswered, however there is growing evidence of genetic variability that modulates sensitivity to, and effects of, vitamin D exposure. It is recognized that 1α,25(OH)_2_D_3_ may have a role in regulating activity of a wide number of genes, directly and through epigenetic changes including at sites directly related to immune cell transcription [36].

## 5. Nuclear Actions, Genetic Polymorphisms and Environmental Interactions

### 5.1. Nuclear Actions of Vitamin D

The vitamin D receptor (VDR), encoded by the *VDR* gene [37], is a member of the nuclear receptor family subgroup NR1I [38]. The VDR mediates its actions by first binding with its ligand (1α,25(OH)2D3) then forming a heterodimer with the retinoid X receptor (RXR) [39]. This heterodimer binds to the VDR response element (VDRE) and initiates recruitment of nuclear proteins into the transcriptional complex [39]. The VDR is found on tissues throughout the body including immune cells, with effects on apoptosis and cell differentiation [37]. The DNA-bound VDR/RXR heterodimers can also down regulate transcription; such is the case with T cell cytokine production [38]. Polymorphisms of the *VDR* gene could, therefore, have significant effects on immune regulation by altering the differentiation and proliferation response [39].

### 5.2. Genetic Polymorphisms in *VDR* and Other Related Genes

Mutation to the *VDR* gene is responsible for conditions such as the rare monogenetic disease 1,25-dihydroxivitamin D resistant rickets, however many more subtle genetic polymorphisms are frequent in the population, although the significance has not been systematically analysed [39]. Multiple studies have reported significant association between polymorphisms in the *VDR* gene with asthma as well as a range of autoimmune conditions, although the findings between cohorts have not been uniform [37,40,41]. Studies of several major North American cohorts identified a number of single nucleotide polymorphisms (SNP) that conferred an increased risk of asthma, including ApaI [37]. Of interest, the *ApaI* C allele was associated with and increased risk of asthma (over represented in asthma cases) from the Nurses’ Health Study (NHS) and a Canadian cohort [37], whereas the same allele was protective in the Childhood Asthma Management Program (CAMP) [40]. It is possible this discrepancy is influenced by differences in age, atopic risk and gender between study populations. In addition to this inconsistence in the direction of risk, other studies have failed to show any associations between *VDR* polymorphisms and asthma or the expression of related allergic phenotypes such as eosinophilia and changes in total IgE level [41]. More recently, in a series of genes that were albeit weakly associated with allergy phenotypes, *VDR* polymorphisms were associated with both asthma and atopy [42]. 

Another gene of interest is *VDBP* encoding for the vitamin D-binding protein (VDBP). This is highly polymorphic (>120 variants) and displays racial and geographic trends. In addition to transporting vitamin D in circulation, VDBP has anti-inflammatory and immunomodulatory functions including effects on macrophage activation. Polymorphisms that influence the binding affinity and immune functions may partly explain the differences in 25(OH)D status and prevalence of certain disease (e.g., autoimmune disease, allergy) observed geographically and between individuals of different races [43]. In their review on the vitamin D axis in lung disease Chisimba *et al.* summarise inconsistent findings on the relationship of *VDBP* polymorphisms in the development of asthma, although the immunomodulatory role of VDBP makes it a sensible target of further investigation in allergic conditions. 

Contradictions and inconsistencies in findings between populations are not uncommon in genetic studies, and are likely to reflect the complex, multifactorial and heterogeneic nature of the allergic/asthma phenotypes, involving multiple and variable genetic and environmental pathways.

### 5.3. Gene-Environment Interactions

A likely reason that genetic association studies have been largely conflicting and failed to identify specific ‘causal’ genes is that the polymorphisms do not necessarily influence risk directly, but may more directly reflect the effect of environmental exposures [44]. Some relationships between genotype and disease will only be seen in conditions of “high” exposure to an environmental factor of interest, whereas others may only be seen in conditions of “low” exposure. Thus, failure to examine the relationship in the context of exposure could lead to an inaccurate interpretations or missing potential relationships. Similarly, the potential effects of environmental exposures on health outcomes could be missed if variations in genetic predisposition are not taken into account. These effects are likely to explain inconsistencies between studies looking at the links between genetics and asthma phenotype. Functional consequences of *VDR* polymorphisms may, therefore, vary with the availability of vitamin D to tissues and become apparent, or exaggerated, in an environment of vitamin D deficiency, such as observed in the case of several infectious diseases [45,46]. Similar complex interactions are now recognised with other genes and other environmental exposures in the context of allergic disease, including functional genetic polymorphisms that are modified by the effects of microbial exposure [47] and cigarette smoke exposure [48]. All of these observations provide further evidence that allergic disease has complex multifactorial etiology, with numerous (and diverse) predisposing genes and a wide range of interacting environmental factors [44]. The lack of linear associations between many functional polymorphisms and disease risk, due to these complex gene-environmental interactions, will continue to provide a major challenge in unraveling the pathogenesis of allergic disease.

## 6. Pregnancy

During pregnancy the foetus is exposed to vitamin D through cord blood supply and the ability of 25(OH)D and 24,25(OH)D to cross the placenta [23]. Maternal vitamin D intake during pregnancy (assessed by food frequency questionnaires), is reported to be inversely associated with wheeze [49,50,51], allergic rhinitis and asthma [6]. Miyake *et al*. found vitamin D to be inversely associated with wheeze in Japanese children aged 16–24 months, an association that was no longer significant when adjusted for docosahexaenoic acid intake [51]. This reflects the relevance of dietary confounders and the differences in intake patterns between populations. In Japan, a major source of dietary vitamin D is fish which is a rich source of n-3 fatty acids, whereas other countries such as the US rely heavily on vitamin D fortified milk [51] which is relatively high in saturated, and low in polyunsaturated, fatty acids [52]. Wheezing is not necessarily indicative of allergic disease as we know that wheeze may be induced by viral infection [53]. While vitamin D may be associated with the development of wheeze through other mechanisms (see Section 4.3) these reports of wheeze are not necessarily related to a true Th2-dominant allergic response. Camargo *et al.* [49,50] conducted a similar study previous to Miyake *et al.* [51] and have published results of their 2 and 3 year follow-ups. Basing their “recurrent wheeze” classification on the Asthma Predictive Index, the authors report a significant inverse association between antenatal dietary and supplemental vitamin D intake and the risk of ever having wheeze or having recurrent wheeze symptoms. For each 2.5 µg/day (100 IU) incremental increase in vitamin D intake the authors found a 10% decrease risk of wheeze and 13% decrease risk of recurrent wheeze at 2 years (OR 0.90; 95% CI, 0.83–0.97 and OR 0.87; 95% CI, 0.77–0.97, respectively) and similar risk of recurrent wheeze at 3 years of age (OR 0.80; 95% CI 0.72–0.90), adjusted for multiple factors. The association remained significant after adjusting for several predisposing risk factors and dietary components such as calcium, vitamin E, fish and vegetable intake. However, there was no association between maternal vitamin D and eczema risk at 3 years. Similar results have been reported by Erkkola *et al.* [6] who found lower rates of both allergic rhinitis (OR 0.85; 95% CI 0.75–0.96, *p* < 0.01) and asthma (OR 0.80; 95% CI 0.65–0.98, *p* < 0.05) in 5 year old children with higher maternal vitamin D intake in pregnancy, with no relationship to eczema (OR 0.95; 95% CI 0.86–1.05, *p* > 0.05). The cohort studied by Camargo *et al.* [49,50] involved children at high-risk of asthma and allergy, with all participants having eczema themselves or maternal history of asthma. We have found similar results to Camargo *et al.* in terms of eczema outcomes in high-risk infants [54]. There was no association between vitamin D intake from diet or supplements during pregnancy, with the risk of eczema in infants at 12 months of age. There are several genetic and environmental differences between the groups such as the predisposition to type 1 diabetes [6] or allergy [50] and locations (Boston, USA [51], Finland [6]) that must be considered when comparing results. However, in general dietary/supplemental intake data neglects the contribution of UV radiation to physiological status. For this reason serological measures of 25(OH)D may be considered preferable as it reflects the combination of endogenous and exogenous sources. 

Gale *et al.* recorded serum 25(OH)D in addition to dietary and supplemental vitamin D intake and estimated UV exposure in 466 pregnant women in the UK [55]. In this cohort 6.5% of women took a vitamin D-containing supplement, a factor that was borderline significant for vitamin D status, and only 3 women met the UK dietary recommendation of 10 µg/day. UV exposure was more strongly related to serum 25(OH)D with a correlation coefficient of 0.60 (*p* < 0.001). The authors report that the risk of atopy was significantly increased in offspring of mothers in the highest quartile of serum 25(OH)D. Compared to the first quartile, the highest quartile of maternal serum 25(OH)D represented an OR of 3.26 (95% CI 1.15–9.29) for eczema at 9 months of age, and 5.40 (95% CI 1.09–26.65) for asthma at 9 years. As UV exposure was the dominant factor in 25(OH)D status this paper provides different information to those focussing on oral intake. To our knowledge this is the only paper that reports such an association between maternal 25(OH)D and atopy. A recent paper by Morales *et al.* [56] involving >1200 Spanish children found no association between maternal serum 25(OH)D during pregnancy and wheezing at 1 or 4 years, or asthma at 4–6 years, although the highest quartile of maternal serum 25(OH)D was protective of lower respiratory tract infections by age 1 year. Cord blood (CB) has been used as a measure of fetal vitamin D status at birth in the investigation of immune function and allergic outcomes. However, it is recognized that CB 25(OH)D concentrations are consistently lower than maternal levels [23] and may also be lower than infant levels [57]. In a longitudinal study from birth to five years Rothers *et al.* examined CB 25(OH)D in relation to total and allergen-specific IgE, sensitization, allergic rhinitis and asthma in a predominantly Caucasian (76.7%) population [58]. The authors report that both low (<50 nmol/L) and high (>100 nmol/L) CB concentrations were associated with total (coefficient 0.27; 95% CI 0.08–0.47, *p* = 0.007 and coefficient 0.27; 95% CI 0.00–0.54, *p* = 0.054, respectively) and allergen specific IgE (OR 2.8; 95% CI 1.2–6.6, *p* = 0.02 and OR 3.6; 95% CI 1.2–10.5, *p* = 0.02, respectively), while high concentration was associated with increased risk of sensitisation (OR 3.4; 95% CI 1.0–11.4, *p* = 0.046). These findings are somewhat supported by a Swedish study involving predominantly Caucasian subjects that reported CB 25(OH)D to be positively correlated with tolerogenic cytokine IL-10 and a higher ratio of IL-10 to IgE [59]. The authors do not report the U-shaped curve observed by Rothers *et al.* [58] but rather a linear correlation of *r* = 0.21 for serum 25(OH)D and IL-10 [59]. In addition, similar findings to that of Rothers *et al.* [58] have been reported in Caucasian adults, whereby IgE levels were lowest in individuals with serum 25(OH)D 100–125 nmol/L but significantly elevated for 25(OH)D concentrations <25 nmol/L (OR 1.29; 95% CI 1.09–1.48) and >135 nmol/L (OR 1.56; 95% CI 1.17–1.95) [60].

In a seperate population (inner city birth cohort, 71% black, 20% Hispanic, 91% with at least one atopic parent) Chi *et al.* investigated CB 25(OH)D and cytokine responses [61]. This study found no significant correlation between CB 25(OH)D and IL-10. Interferon-γ response to LPS were however positively correlated with CB 25(OH)D (*r* = 0.11, *p* = 0.01), while there appeared to be a negative correlation between CB 25(OH)D and the proportion of Treg cells. Recently, genetic traits have been identified in African populations that explain part of the predisposition to asthma and the related cytokine responses [62]. This adds evidence that genetic variation influences both nuclear and non-nuclear actions and helps explain variations in immune function and allergy trends between population groups. Genetics and the *in utero* environment do not tell the whole story of allergy development and it is likely that there are distinct postnatal effects that must be considered.

## 7. Infancy and Childhood

The evidence to date suggests that adequate or higher concentrations of 25(OH)D in childhood are generally protective of allergic disease [63,64,65], although not all reports agree [66,67]. So far the main focus of those studies has been on respiratory allergic outcomes such as allergic rhinitis, asthma and inhalant allergen sensitization. Members of our group have reported lower serum 25(OH)D in Qatari children with asthma compared to controls (43.1 ± 27.5 nmol/L and 67.1 ± 24.7 nmol/L, respectively, *p* < 0.001) [65]. In this case-control study serum 25(OH)D <50 nmol/L was the strongest predictor of asthma (OR 4.82, *p* < 0.001) among variables including family history of asthma (OR 2.45, *p* < 0.001) and serum IgE levels (OR 1.98, *p* = 0.003). Investigating clinical outcomes without immune function data, Camargo *et al.* [67] report a linear relationship between CB 25(OH)D with wheeze at age 15 months through 5 years and respiratory tract infection at 3 months. The authors report no association between CB 25(OH)D and asthma or sensitization. Conducted in Wellington and Christchurch, New Zealand, the birth cohort (*n* = 922) comprised 71% European participants, and provides valuable information due to the prospective cohort design, compared to cross-sectional design of other studies [64,65,66]. Cross-sectional studies may be subject to reverse causality, for example, where individuals with respiratory conditions such as asthma or allergic rhinitis avoid outdoor activities and subsequently receive less UVB exposure. 

In one of the largest cross-sectional studies in the USA (*n* = 18,224) researchers reported a positive association between allergic rhinitis serum 25(OH)D concentration (across quartiles), although there were no consistent relationships between 25(OH)D concentration and sensitization to any particular allergen [66]. This association was most apparent in younger individuals (<20 years of age) with a higher risk of allergic rhinitis in the upper (third and fourth) quartiles of serum 25(OH)D (OR 3.12; 95% CI 1.11–8.81, *p* = 0.032, and OR 4.05; 95% CI 1.45–11.28, *p* = 0.008, respectively). While the same relationship was seen in the >20 years age group (for the highest quartile) it was not as strong (OR 1.25; 95% CI 1.03–1.53, *p* = 0.024).

These findings were not reproduced in a more recent USA population study where the prevalence of allergic rhinitis did not vary with 25(OH)D concentration, while there was a protective relationship with sensitisation in children and adolescents [64]. Both Wjst and Hyppönen [66] and Sharief *et al.* [64] have well powered studies backed by biological measures and clinical outcome data, however do not specifically address early life exposure and are limited by the cross-sectional design. Data is not available for neonates or young children and thus may overlook a crucial period in the aetiology of allergic disease. The average age for the children/adolescents group in the paper by Sharief *et al*. [64] was 12.5 years, well beyond the critical early period of immune development [68]. 

Longitudinally, an Australian study has shown a protective relationship between serum 25(OH)D levels at age 6 years and subsequent development of allergic rhinitis at age 14 [63]. In this non-selected community cohort there was no relationship between 25(OH)D levels and “current” allergic rhinitis, although serum 25(OH)D concentration at 6 years was protective of allergic rhinitis at 14 years (OR 0.17; 95% CI 0.05–0.59, *p* = 0.005). As the majority of asthma is diagnosed by 6 years of age these findings do not present relevance to asthma development *per se*, but possibly on the persistence of the condition into adulthood. 

Fewer studies have investigated vitamin D status and food allergy outcomes. The study by Sharief *et al.* [64] found that lower serum 25(OH)D (of ≤37.5 nmol/L) was associated with both peanut (OR 2.39; 95% CI 1.29–4.45, *p* = 0.005) and shrimp (OR 3.08; 95% CI 1.16–8.19, *p* = 0.02) sensitization, however, other studies of food allergy are largely limited to epidemiological data without serological measures of vitamin D [69,70,71,72]. With the global increase in food allergy globally [11] this is a priority area for future research.

A number of longitudinal observational studies have specifically examined infant vitamin D supplementation in the context of allergic disease. This data has the advantage of focussing on the early life period and may therefore provide insight into the aetiological role of vitamin D. Despite the observed protective association of serum 25(OH)D and allergy, it has been noted that the widespread practice of supplementation coincided with the rise in allergic disease [35]. Supporting this observation are several studies which found vitamin D supplementation to be associated with an increased risk of atopic dermatitis [73], allergic rhinitis [35,74], asthma, sensitisation [69,74] and food allergy [70], however none of these papers are supported by biological measures of vitamin D status. Hyponnen *et al.* [74] reported that supplementation with 50 µg vitamin D/day (2000 IU) in the first year of life was associated with a higher risk of allergic rhinitis (OR 1.66; 95% CI 1.1–1.6, *p* < 0.001) and atopy (OR 1.46; 95% CI 1.4–2.0, *p* < 0.001). The dose cited in this paper (50 µg/day or 2000 IU) is substantially higher than the 10 µg/day 400 IU recommended for infants today by the American Academy of Pediatrics (AAP) [75]. However, other authors investigating infant intakes in this lower dose range (closer to AAP recommendations) have also shown that atopic manifestations (particularly atopic dermatitis) were more prevalent in children with “high” (>13.1 μg/day) *versus* “low” (≤13.1 μg/day) intake of vitamin D [73]. The increase in relative risk of atopic dermatitis (AD) was 18% per 1 µg/day increase in vitamin D intake, and a high vitamin D intake was a stronger predictor of AD than was family history of atopy. The papers by Bäck *et al.* [73] and Hyppönen *et al.* [74] were based on Swedish and Finish populations, respectively; countries that share similar environmental conditions such as climate and latitude. There is also some evidence that the effect of supplementation may depend on whether vitamin D is administered in a fat- or water-soluble form. 

Kull *et al.* [70] demonstrated that children given vitamin D (400 IU) in water-soluble form during the first year of life had an almost two-fold increase in asthma (OR 2.18; 95% CI 1.45–3.28), food allergy (OR 1.89; 95% CI 1.33–2.65), and sensitization (OR 1.88; 95% CI 1.34–2.64) at 4 years of age compared with children having received the same vitamins in peanut oil (although there was no difference in the risk of allergic rhinitis or eczema). These contrasting results highlight the need for randomised controlled trials (RCT) to provide clearer picture of role of vitamin D during infancy in regulation of allergic disease, including uptake, metabolism and immunomodulation.

In summary, the contrary epidemiological observations between serum status (generally protective) and oral exposure (supplementation increasing allergy risk in many studies) highlight the uncertainty surrounding vitamin D in allergy development and precludes population recommendations. Most of these observational studies did not have direct serological measures of 25(OH)D levels to accurately confirm vitamin D status and cross sectional study designs involving older children and adults fail to provide evidence on aetiological factors. Although the significance is not clear, the findings show that the timing and route of exposure may play a critical role in immune function. Future research must accurately account for the different sources of vitamin D, stage of immune development and genetic factors in order to understand the immunological consequences of changing vitamin D status through supplementation or lifestyle changes. 

## 8. Conclusions and Future Directions

A high percentage of pregnant women globally are known to have 25(OH)D concentrations in the insufficient and deficient ranges [22,76,77] yet currently very little information is available on the impact of this deficiency on neonatal immune function and subsequent risk of allergic disease. There is much evidence to show vitamin D affects differentiation and proliferation of immune cells [2,24,25] and that genetic polymorphisms, including of *VDR* and *VDBP*, may contribute to disease susceptibility [42,78,79,80,81]. As this early life stage is relevant to programming of future immune function it is crucial to understand the effects of vitamin D and identify optimal status levels. Future research should differentiate oral intake from endogenous contributions to 25(OH)D status in order to clarify the immunological effects of each. Randomized control trials are ultimately required to determine the effects of vitamin D supplementation on the developing immune system, and any potential role in preventing allergic disease.
